# Refractory out-of-hospital cardiac arrest with ongoing cardiopulmonary resuscitation at hospital arrival – survival and neurological outcome without extracorporeal cardiopulmonary resuscitation

**DOI:** 10.1186/s13054-018-2176-9

**Published:** 2018-09-29

**Authors:** Emilie Gregers, Jesper Kjærgaard, Freddy Lippert, Jakob H. Thomsen, Lars Køber, Michael Wanscher, Christian Hassager, Helle Søholm

**Affiliations:** 1 Department of Cardiology 2142, The Heart Centre, Copenhagen University Hospital Rigshospitalet, Blegdamsvej 9, 2100 Copenhagen O, Denmark; 2 Emergency Medical Services, Copenhagen, Denmark; 3 Department of Cardiothoracic Anaesthesia 4142, The Heart Centre, Copenhagen University Hospital Rigshospitalet, Copenhagen, Denmark; 4 Department of Cardiology, Zealand University Hospital Roskilde, Roskilde, Denmark

**Keywords:** Cardiac arrest, Ongoing CPR, Refractory cardiac arrest, Survival

## Abstract

**Background:**

The prognosis in refractory out-of-hospital cardiac arrest (OHCA) with ongoing cardiopulmonary resuscitation (CPR) at hospital arrival is often considered dismal. The use of extracorporeal cardiopulmonary resuscitation (eCPR) for perfusion enhancement during resuscitation has shown variable results. We aimed to investigate outcome in refractory OHCA patients managed conservatively without use of eCPR.

**Methods:**

We included consecutive OHCA patients with refractory arrest or prehospital return of spontaneous circulation (ROSC) in the Copenhagen area in 2002–2011.

**Results:**

A total of 3992 OHCA patients with resuscitation attempts were included; in 2599, treatment was terminated prehospital, and 1393 (35%) were brought to the hospital either with ROSC (*n* = 1285, 92%) or with refractory OHCA (*n* = 108, 8%). Of patients brought in with refractory OHCA, 56 (52%) achieved ROSC in the emergency department. There were no differences between patients with refractory OHCA or prehospital ROSC with regard to age, sex, comorbidities, or etiology of OHCA. Time to emergency medical services (EMS) arrival was similar, whereas time to ROSC (when ROSC was achieved) was longer in refractory OHCA patients (EMS, 6 (5–9] vs. 7 [5–10] min, *p* = 0.8; ROSC, 15 [9–22] vs. 27 [20–41] min, *p* < 0.001). Independent factors associated with transport with refractory OHCA instead of prehospital termination of therapy were OHCA in public (OR, 3.6 [95% CI, 2.2–5.8]; *p* < 0.001), witnessed OHCA (OR, 3.7 [2.0–7.1]; *p* < 0.001), shockable rhythm (OR, 3.0 [1.9–4.7]; *p* < 0.001), younger age (OR, 1.2 [1.1–1.2]; *p* < 0.001), and later calendar year (OR, 1.4 [1.2–1.6]; *p* < 0.001). Thirty-day survival was 20% in patients with refractory OHCA compared with 42% in patients with prehospital ROSC (*p* < 0.001). Four of 28 refractory OHCA patients with duration of resuscitation > 60 min achieved ROSC. No difference in favorable neurological outcome in patients surviving to discharge was found (prehospital ROSC 84% vs. refractory OHCA 86%; *p* = 0.7).

**Conclusions:**

Survival after refractory OHCA with ongoing CPR at hospital arrival was significantly lower than among patients with prehospital ROSC. Despite a lower survival, the majority of survivors with both refractory OHCA and prehospital ROSC were discharged with a similar degree of favorable neurological outcome, indicating that continued efforts in spite of refractory OHCA are not in vain and may still lead to favorable outcome even without eCPR.

## Background

Survival after out-of-hospital cardiac arrest (OHCA) has increased in recent years, and about one-fifth of patients achieve return of spontaneous circulation (ROSC) at hospital arrival, but only approximately 10% achieve long-term survival [1, 2]. Factors such as bystander cardiopulmonary resuscitation (CPR), early defibrillation, and emergency medical services (EMS) response time have been proven as important prognostic factors for both short-term and long-term survival for OHCA patients as well as favorable neurologic outcome after hospital discharge [1, 3, 4].

Studies have found shorter time to ROSC to be vital for survival, with more than 50% survival when ROSC was achieved in less than 5 min after EMS arrival compared with approximately 10% when ROSC was achieved after 25 min [5, 6]. Despite the best efforts, not all patients achieve ROSC in the prehospital setting; under these circumstances, the OHCA is categorized as refractory [7, 8].

The prognosis in refractory OHCA has previously been shown to be dismal [5, 6, 9]. Attempts at improving outcome are ongoing, and the use of extracorporeal cardiopulmonary resuscitation (eCPR) for perfusion enhancement during resuscitation has been implemented on a trial basis in different countries for both in-hospital and prehospital treatment of refractory cardiac arrest [10]; however, observational studies on the use of eCPR have shown variable results [7, 11–16]. No randomized studies have yet been completed, though some randomized trials are underway investigating the use of eCPR in the emergency department (ED) and in the prehospital setting, respectively [17, 18].

In an attempt to establish a baseline for such studies in patients with refractory OHCA, we aimed to investigate survival and neurological outcome in a consecutive clinical cohort of patients with refractory OHCA managed conservatively at university hospitals without the use of eCPR.

## Methods

### Patients and study area

Consecutive patients with OHCA were included in the study from June 2002 through 2011. Adult patients older than 18 years of age in the Capital Region of Denmark with OHCA were included. Patients with obvious signs of death (e.g., rigor/livor mortis) and non-Danish residents (owing to unavailable outcome data) were excluded from the study.

All patients were treated by EMS consisting of an emergency ambulance with basic life support equipment and a defibrillator and a response unit in a separate vehicle staffed with a paramedic and an attending physician (anesthesiologist). The response unit had mechanical CPR with the AutoPulse® Resuscitation System (Zoll Medical, Chelmsford, MA, USA) available for use in case of OHCA when deemed appropriate. The EMS are dispatched to all patients with presumed OHCA with the treatment protocol according to current advanced life support guidelines of the European Resuscitation Council. If ROSC was not achieved in the prehospital setting, the EMS physician could decide to either terminate active therapy or transport the patient to the nearest hospital with ongoing CPR (termed *refractory OHCA*) when the OHCA was considered potentially reversible based on an overall clinical assessment. This procedure is in accordance with current guidelines [19, 20]. Patients with rearrest after obtaining ROSC were classified as EMS-witnessed OHCA. The attending EMS physician used an Utstein-style registration sheet as documentation, on which prehospital data were registered immediately after the end of each case [21]. Patients with refractory OHCA or rearrest in the hospital were treated by a resuscitation team that collaborated in the decision of continuing vs. terminating treatment. The team included an anesthesiologist and a specialist in cardiology or internal medicine.

### Postresuscitation care

Patients were admitted to the nearest hospital, either one of two university heart centers or one of six nontertiary university hospitals. In case of signs of ST segment elevation myocardial infarction (STEMI), the patient was admitted to a heart center for acute coronary angiography (CAG). In case of ROSC, all comatose patients were subsequently treated in an intensive care unit (ICU). Patients could be referred to a heart center for advanced treatment at any time during the hospital stay. A single investigator reviewed all individual patient charts with a focus on in-hospital postresuscitation care.

In-hospital treatment was terminated if prospect of recovery was not present on the basis of clinical assessment and parameters such as burden of comorbidities, echocardiogram, electroencephalography (EEG), inability to get off ventilator, and blood test results.

### Data collection

By using the personal identification number provided to all Danish residents, we linked the OHCA cohort to the Danish Civil Registration Registry, which holds vital status on all Danish citizens, and primary outcome of the current study (30-day mortality) was obtained. All diagnoses and surgical procedures from all hospital admissions are continually registered in the National Patient Registry. On the basis of obtained data on comorbidity, coexisting conditions, and surgical procedures, we calculated the Charlson comorbidity Index (CCI) [22]. In addition, neurological outcome at hospital discharge was assessed from patient charts with the Cerebral Performance Categories scale (CPC). Favorable neurological outcome was defined as CPC 1 or 2, nonfavorable as 3–4, and dead as CPC 5. Data on termination of active therapy was also acquired from patient charts.

### Statistics

Data are presented as mean ± SD; as median and quartiles (Q1 and Q3); and, for categorical variables, as number and percent. Differences were analyzed with Student’s unpaired *t* test, Wilcoxon rank-sum test, or chi-squared test as appropriate. Logistic regression analyses were performed to test for multivariate factors associated with transport with ongoing CPR instead of prehospital termination of active therapy estimating OR and 95% CI; age (per 5 years), sex, public OHCA, witnessed OHCA, bystander CPR, calendar year, and initial rhythm were included in the multivariate analysis. Mortality is presented with Kaplan-Meier curves, and differences were tested using the log-rank test. Univariate and multivariate proportional hazards regression analyses (Cox regression) were performed estimating HRs and 95% CIs, adjusting for potential confounders after checking for the underlying assumptions of proportionality and lack of interactions; refractory OHCA, age, public OHCA, witnessed OHCA, bystander CPR, resuscitation length > 30 min, calendar year, and high comorbidity burden (CCI ≥ 3) were included in the multivariate analysis. All statistical analyses were performed using SAS version 9.4 software (SAS Institute, Cary, NC, USA) with level of significance defined as *p* < 0.05.

## Results

### Patient characteristics

**Fig. 1:**
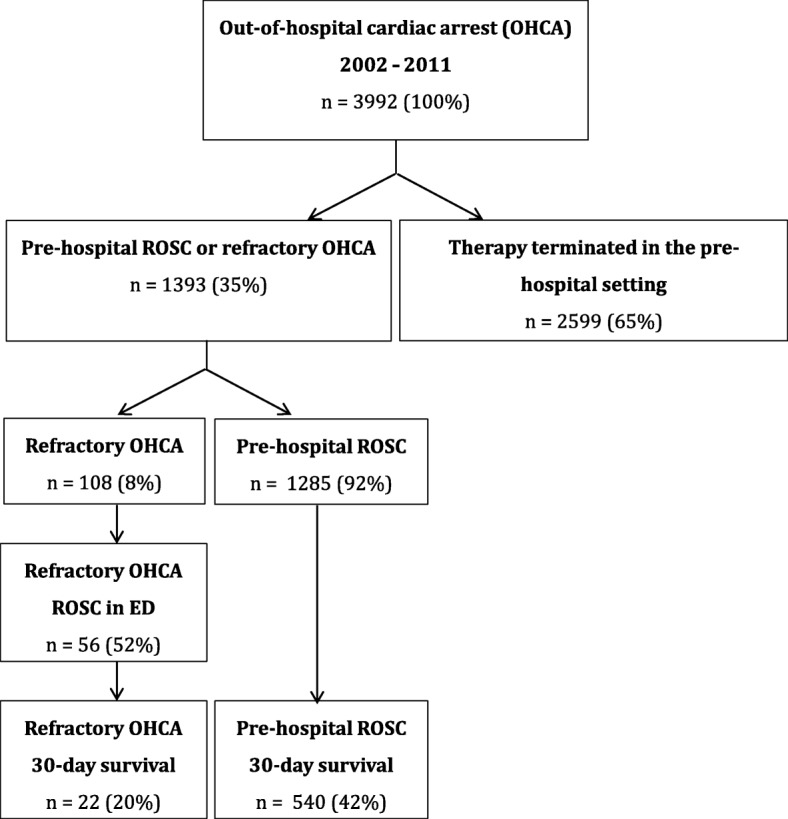
Flowchart of adult out-of-hospital cardiac arrest patients included consecutively in the study during the 9-year study period. *ED* Emergency department, *OHCA* Out-of-hospital cardiac arrest, *ROSC* Return of spontaneous circulation

A total of 3992 OHCA patients underwent attempted resuscitation in the study period, of whom 1393 (35%) were brought to the hospital either with successful resuscitation (*n* = 1285, 92%) or refractory OHCA (*n* = 108, 8%) (Fig. 1). Of patients with refractory OHCA (defined as patients transported to hospital with ongoing CPR), 56 (52%) had ROSC in the ED.

**Fig. 2:**
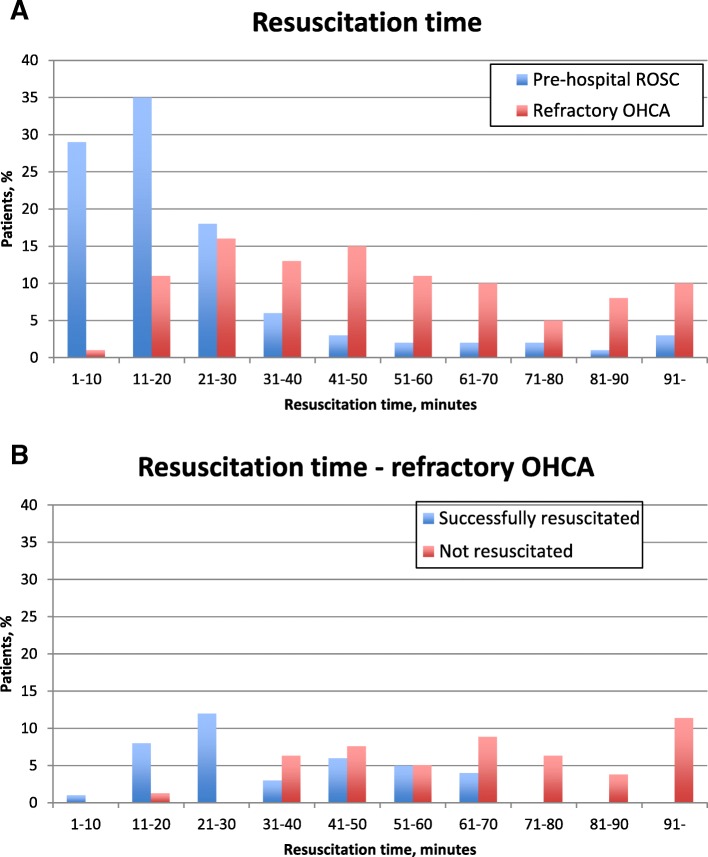
Distribution of duration of resuscitation in out-of-hospital cardiac arrest (OHCA) patients separated as prehospital return of spontaneous circulation (ROSC) and refractory OHCA patients (**a**) and refractory OHCA patients only separated as successful and unsuccessful resuscitation (**b**)

Patient characteristics can be found in Table 1. There were no statistically significant differences in age, sex, or cardiovascular comorbidity between the groups of patients with refractory OHCA or prehospital ROSC. Fewer patients with refractory OHCA had OHCA in a public setting and OHCA witnessed by the EMS compared with patients with prehospital ROSC. Time to EMS arrival was similar: 6 min (5–9) and 7 min (5–10), respectively. Time to ROSC was longer in refractory OHCA patients achieving ROSC than in prehospital ROSC patients (median 27 min [Q1–Q3, 20–41] vs. 15 min [9–22]). Duration of the resuscitation attempt in refractory OHCA patients where resuscitation was not successful was 65 min (46–85). The distribution of resuscitation time in patients with refractory OHCA and prehospital ROSC can be seen in Fig. 2a, whereas duration of resuscitation in successful vs. unsuccessful resuscitation of refractory OHCA patients can be seen in Fig. 2b. Fifteen of 49 (31%) patients with refractory OHCA and duration of resuscitation > 40 min achieved ROSC, and 4 of 28 (14%) patients with refractory OHCA and duration of resuscitation > 60 min achieved ROSC (Fig. 2b). There was no difference in the share of patients awake (Glasgow Coma Scale score > 9) after ROSC between refractory OHCA patients and prehospital ROSC patients.

**Table 1:** Patient characteristics, prehospital data, and postresuscitation care data in the out-of-hospital cardiac arrest study population

	Total *n* = 1393	Prehospital ROSC *n* = 1285 (92%)	Refractory OHCA *n* = 108 (8%)
Age, years (mean ± SD)	65 ± 15	65 ± 11	61 ± 17
Sex (male), *n* (%)	961 (70%)	882 (70%)	79 (77%)
Cardiovascular comorbidity, *n* (%)			
- Chronic ischemic heart disease	289 (21%)	265 (21%)	24 (24%)
- Congestive heart failure	215 (16%)	203 (16%)	12 (12%)
- Type 2 diabetes	164 (12%)	152 (12%)	12 (12%)
- Hypertension	494 (36%)	460 (37%)	34 (33%)
- Hypercholesterolemia	252 (19%)	233 (19%)	19 (19%)
- Active smoking	344 (25%)	324 (26%)	20 (20%)
High comorbidity burden (CCI ≥ 3)^a^, *n* (%)	273 (20%)	252 (20%)	21 (21%)
OHCA circumstances			
- Shockable primary rhythm, *n* (%)	704 (52%)	652 (52%)	52 (50%)
- Public^b^, *n* (%)	496 (37%)	443 (36%)	53 (52%)
- Witnessed OHCA, *n* (%)	1126 (86%)	1040 (86%)	86 (85%)
◦ By EMS^b^	70 (7%)	58 (6%)	12 (14%)
- Bystander CPR, *n* (%)	704 (55%)	649 (55%)	55 (56%)
- Time to EMS (min), median (Q1-Q3)	7 (5–9)	6 (5–9)	7 (5–10)
- Time to ROSC (min), median (Q1-Q3)^b^	15 (9–23)	15 (9–22)	27 (20–41)
- Length of resuscitation attempt (min), median (Q1-Q3)^b^	17 (10–29)	16 (9–26)	45 (27–68)
- Time to emergency room (min), median (Q1-Q3)^b,c^	40 (31–52)	40 (32–52)	35 (25–47)
OHCA etiology			
- Cardiovascular etiology, *n* (%)	1100 (81%)	1012 (81%)	88 (87%)
◦ STEMI, *n* (%)	313 (28%)	293 (29%)	20 (23%)
◦ NSTEMI, *n* (%)	229 (21%)	218 (22%)	11 (13%)
◦ Cardiogenic shock, *n* (%)	63 (6%)	54 (5%)	9 (10%)
◦ Primary arrhythmia, *n* (%)	166 (15%)	157 (16%)	9 (10%)
◦ Pulmonary embolism, *n* (%)^b^	21 (2%)	15 (1%)	6 (7%)
◦ Other cardiovascular etiology, *n* (%)^b^	308 (28%)	275 (27%)	33 (38%)
Admitted to heart center^b^	836 (61%)	763 (61%)	73 (71%)
In-hospital^d^			
- GCS > 9 after ROSC, *n* (%)	127 (11%)	123 (11%)	4 (7%)
- Admitted ICU, *n* (%)^b^	1085 (90%)	1029 (89%)	56 (100%)
- ICU days, median (Q1-Q3)^b^	4 (2–7)	4 (2–7)	7 (4–9)
- Mechanical ventilation, *n* (%)^b^	1085 (90%)	1029 (89%)	56 (100%)
- TTM, *n* (%)	656 (55%)	628 (56%)	28 (51%)
- IABP, *n* (%)^b^	48 (4%)	40 (3%)	8 (14%)
- EEG, *n* (%)	217 (18%)	203 (18%)	14 (25%)
- CT cerebrum, *n* (%)	435 (36%)	414 (36%)	21 (38%)
- Thrombolysis, *n* (%)	9 (1%)	8 (1%)	1 (2%)
- CAG, *n* (%)	500 (41%)	480 (42%)	20 (36%)
◦ Early CAG (< 24 h)	377 (31%)	359 (31%)	18 (32%)
- Revascularization (of patients with CAG)			
◦ PCI, *n* (%)	269 (54%)	259 (54%)	10 (50%)
◦ CABG, *n* (%)	53 (11%)	51 (11%)	2 (10%)
- Temporary pacemaker, *n* (%)	63 (5%)	58 (5%)	5 (9%)
- Permanent pacemaker/ICD, *n* (%)	201 (17%)	194 (17%)	7 (13%)
Termination of in-hospital treatment^d,e^			
- Anoxic brain damage, *n* (%)^b^	377 (50%)	358 (52%)	19 (25%)
- Circulatory failure, *n* (%)^b^	292 (39%)	239 (36%)	53 (70%)
- Organ failure, *n* (%)	81 (11%)	77 (11%)	4 (5%)
- Seizures, *n* (%)^b^	56 (7%)	56 (8%)	0 (0%)
- High burden of comorbidities, *n* (%)^b^	152 (20%)	148 (22%)	4 (5%)
- Time to ROSC, *n* (%)^b^	35 (5%)	25 (4%)	10 (13%)
- Living will, *n* (%)	9 (1%)	9 (1%)	0 (0%)
At hospital discharge			
- LVEF > 35%, *n* (%)	451 (55%)	430 (55%)	21 (51%)
- Favorable neurological outcome (CPC 1 or 2), *n* (%)	456 (84)	437 (84)	19 (90)
- Nonfavorable neurological outcome (CPC 3 or 4), *n* (%)	89 (16%)	86 (16%)	3 (14%)

*Abbreviations: CPR* Cardiopulmonary resuscitation, *ROSC* Return of spontaneous circulation, *OHCA* Out of hospital cardiac arrest, *EMS* Emergency medical services, *Q1-Q3* Interquartile range, *STEMI* ST elevation myocardial infarction, *NSTEMI* Non-ST elevation myocardial infarction, *GCS* Glasgow Coma Scale, *ICU* Intensive care unit, *TTM* Targeted temperature management, *IABP* Intra-aortic balloon pump, *EEG* Electroencephalography, *CT* Computed tomography, *CAG* Coronary angiography, *PCI* Percutaneous coronary intervention, *CABG* Coronary artery bypass grafting, *ICD* Implantable cardioverter defibrillator, *LVEF* Left ventricular ejection fraction, *CPC* Cerebral Performance Categories, *CCI* Charlson comorbidity index

All percentages are calculated after excluding missing data from the denominator

^a^Charlson comorbidity index (CCI), which is a validating index taking the severity of 22 conditions into account. CCI ≥ 3 was used as a marker of significant comorbidity [22]

^b^Indicates significant difference between patients with prehospital ROSC and refractory OHCA (*p* < 0.05)

^c^Time from emergency call to arrival in the emergency room

^d^Patients achieving and/or remaining in ROSC in the emergency department only

^e^More than one reason may be listed per patient

Time from emergency call to ED was 35 min (25–47) in refractory OHCA and 40 min (32–52) in prehospital ROSC (*p* = 0.003). Time from EMS arrival to arrival at the ED was 29 min (19–39) for refractory OHCA and 35 min (27–44) for prehospital ROSC (*p* = 0.003). Of note, the number of missing values were high for this parameter (60% missing in refractory OHCA and 77% missing in prehospital ROSC). No data on specific time spent on scene was available, except that geography of the region with short distances to hospital and a time of transportation of about 10 min made time to ED a rough surrogate measure of time spent on scene. A load-and-go strategy is estimated to have been used in up to 33% (*n* = 14, 65 missing) of refractory OHCA cases, based on the percentage of cases with time from EMS arrival to arrival at the ED < 20 min.

More than 80% of the OHCAs were due to cardiac causes, with acute coronary syndrome being the main cardiac cause (STEMI in 28% of all cardiac cases and non-STEMI in 21%). There was no significant difference in patients with a cardiac cause between prehospital ROSC patients and refractory OHCA patients. In patients with a cardiac cause, no difference between patients with STEMI, non-STEMI, cardiogenic shock, or primary arrhythmia was found between the two groups; however, more patients with refractory OHCA had pulmonary embolus and fewer had other cardiac causes as etiology than among patients with prehospital ROSC (Table 1).

Comparing refractory OHCA patients with OHCA patients where treatment was terminated in the prehospital setting, we found that more patients with refractory OHCA had public OHCA (52% vs. 16%, *p* < 0.001), witnessed OHCA (86% vs. 61%, *p* < 0.001), EMS witnessed OHCA (13% vs. 4%, *p* < 0.001), bystander CPR performed (56% vs. 36%, *p* < 0.001), and shockable primary rhythm (51% vs. 15%, *p* < 0.001). Resuscitation was considered futile because of long period of anoxia (13%), terminal chronic disease (4%), terminal cancer (2%), major trauma not compatible with life (1%), or charring (0.04%) in 19% of cases with treatment terminated in the prehospital setting (44% unknown). We found no difference in the number of patients where cardiac etiology was presumed (77% vs. 75%, *p* = 0.8).

Independent factors associated with transport to hospital with ongoing CPR instead of prehospital termination of resuscitation were OHCA in a public setting (OR 3.7 [95% CI, 2.3–6.0]), witnessed OHCA (OR 4.0 [2.1–7.4]), shockable rhythm (OR 2.9 [1.8–4.5]), younger age (per 5 years, OR 1.2 [1.1–1.2]), and later calendar year (OR 1.4 [1.2–1.6]). Gender and performance of bystander CPR were not associated with transport to hospital (Table 2).

**Table 2:** Univariate and multivariate factors associated with transport to hospital with ongoing cardiopulmonary resuscitation instead of prehospital termination of active therapy

	Univariable		Multivariable	
	OR (95% CI)	*p* Value	OR (95% CI)	*p* Value
Age at OHCA (per 5 years younger)	1.1 (1.1–1.2)	**< 0.0001**	1.2 (1.1–1.2)	**< 0.0001**
Sex (male)	1.8 (1.1–2.8)	**0.01**	1.2 (0.7–1.9)	0.6
Public OHCA	5.3 (3.6–7.8)	**< 0.0001**	3.6 (2.2–5.8)	**< 0.0001**
Witnessed OHCA	4.0 (2.3–7.0)	**< 0.0001**	3.7 (2.0–7.1)	**< 0.0001**
Bystander CPR performed	2.3 (1.6–3.5)	**< 0.0001**	1.3 (0.8–2.0)	0.3
Shockable initial rhythm	5.9 (4.0–8.7)	**< 0.0001**	3.0 (1.9–4.7)	**< 0.0001**
Calendar year	1.2 (1.1–1.3)	**< 0.0001**	1.4 (1.2–1.6)	**< 0.0001**

*p*-values in bold indicate statistical significance. *OHCA* Out-of-hospital cardiac arrest, *CPR* Cardiopulmonary resuscitation

### Postresuscitation care

When comparing postresuscitation care in patients with refractory OHCA with that in prehospital ROSC patients (Table 1), we found no difference in the number of patients receiving targeted temperature management, CAG, early CAG (within 24 h), percutaneous cardiovascular intervention, coronary bypass grafting, or thrombolysis. More patients with refractory OHCA were treated with an intra-aortic balloon pump (3% vs. 14%, *p* = 0.001), and more received mechanical ventilation and were admitted to an ICU (89% vs. 100%, *p* = 0.005). Computed tomography (CT) of the brain and EEG were performed similarly. Likewise, there was no difference in the number of patients treated with temporary pacemaker or permanent pacemaker/implantable cardioverter defibrillator before hospital discharge (Table 1).

### Termination of in-hospital treatment

Reasons for terminating active in-hospital treatment in patients achieving and/or remaining in ROSC in the ED differed between refractory OHCA patients compared with prehospital ROSC patients (Table 1). Significantly more patients with refractory OHCA had circulatory failure, whereas fewer had treatment terminated because of anoxic brain damage, seizures, and high burden of comorbidities. There was no significant difference between patients with treatment terminated because of organ failure or living will.

### Outcomes

**Fig. 3:**
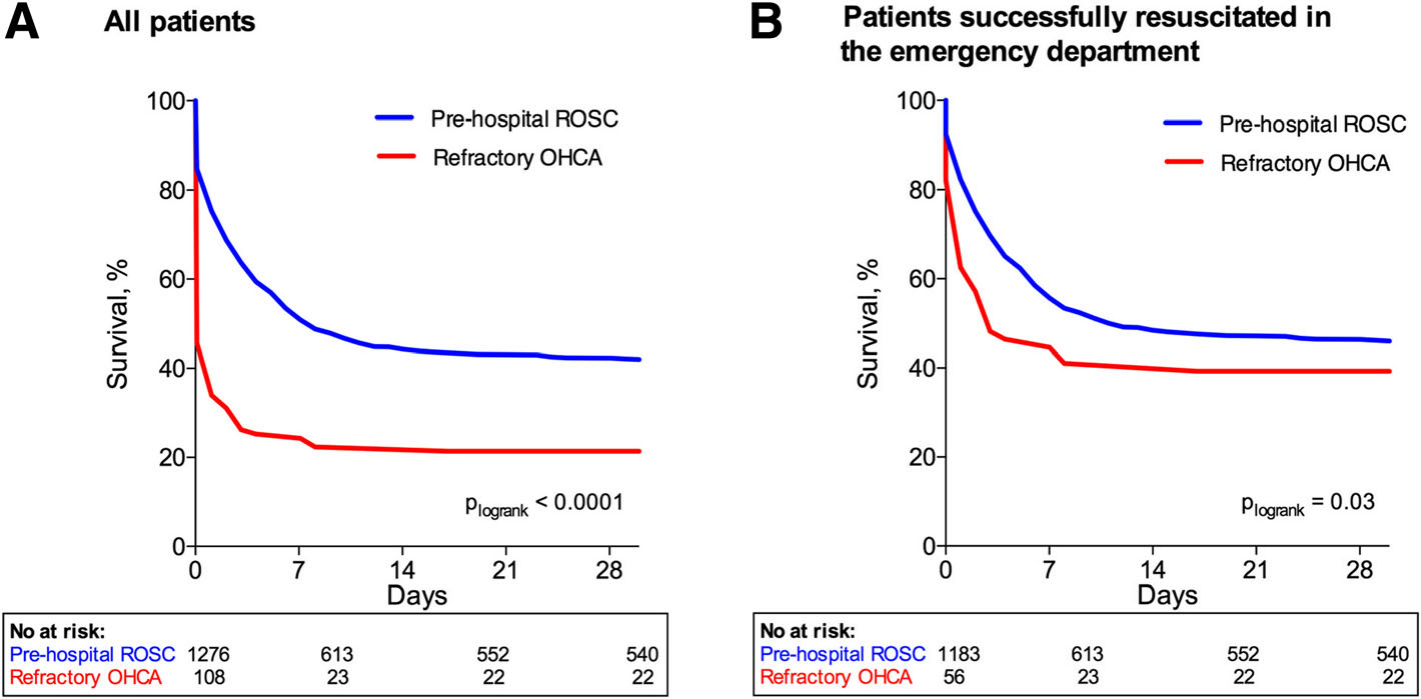
Thirty-day survival in patients with prehospital return of spontaneous circulation (ROSC) and refractory out-of-hospital cardiac arrest (OHCA) divided into all patients (**a**) and patients successfully resuscitated in the emergency department only (**b**)

Survival was significantly lower at all times in refractory OHCA patients than in prehospital ROSC patients; at 24 h after OHCA, survival was 35% vs. 75% (*p* < 0.001); at day 7, it was 28% vs. 60% (*p* < 0.001); and at day 30 after OHCA, survival was 20% (*n* = 22) vs. 42% (*n* = 540) (*p* < 0.001) (Fig. 3a). This was also true when patients with active therapy terminated in the ED were excluded (39% and 46%, respectively, at day 30; *p* = 0.04) (Fig. 3b).

Refractory OHCA, age, public OHCA, witnessed OHCA, bystander CPR performed, high severity of comorbidity, and resuscitation length > 30 min were all found to be independently associated with increased 30-day mortality (Table 3). Calendar year was not associated with 30-day mortality (HR 0.98). Comparing all patients with duration of resuscitation > 30 min with duration < 30 min, we found 30-day survival of 38% and 50%, respectively (*p* < 0.001).

**Table 3:** Univariate and multivariate factors associated with 30-day mortality in out-of-hospital cardiac arrest patients with prehospital return of spontaneous circulation or refractory out-of-hospital cardiac arrest transported to hospital

	Univariable		Multivariable	
	HR (95% CI)	*p* Value	HR (95% CI)	*p* Value
Refractory OHCA	2.2 (1.7–2.7)	**< 0.0001**	2.4 (1.8–3.1)	**< 0.0001**
Age at OHCA (per 5 years older)	1.1 (1.1–1.2)	**< 0.0001**	1.1 (1.1–1.2)	**< 0.0001**
Public OHCA	0.5 (0.5–0.6)	**< 0.0001**	0.7 (0.5–0.8)	**< 0.0001**
Witnessed OHCA	0.7 (0.6–0.9)	**0.0003**	0.6 (0.5–0.8)	**0.0001**
Bystander CPR performed	0.6 (0.5–0.6)	**< 0.0001**	0.6 (0.5–0.8)	**< 0.0001**
Duration of resuscitation attempt > 30 min	1.5 (1.3–1.8)	**< 0.0001**	1.4 (1.2–1.7)	**< 0.0001**
High comorbidity burden (CCI ≥ 3)	1.6 (1.4–1.9)	**< 0.0001**	1.3 (1.1–1.6)	**0.007**
Calendar year	1.0 (1.0–1.0)	0.3	1.0 (1.0–1.0)	0.7

*p*-values in bold indicate statistical significance. *OHCA* Out-of-hospital cardiac arrest, *CPR* Cardiopulmonary resuscitation, *CCI* Charlson comobidity index

Calculating the number needed to treat (NNT), we found that five patients with refractory OHCA need to be transported to hospital with ongoing CPR in order to save one patient with refractory OHCA (NNT 4.9). Including refractory OHCA patients, prehospital ROSC patients, and OHCA patients with prehospital termination of resuscitation in the analysis, NNT was 7.4. Looking only at refractory OHCA patients and OHCA patients with prehospital termination of resuscitation, NNT was 121.

No significant difference in neurological outcome in patients surviving to discharge was found, with 84% of prehospital ROSC patients and 86% of refractory OHCA patients being discharged with a favorable neurological outcome (*p* = 0.7). Resuscitation time > 30 min was not associated with neurological outcome when including all survivors (OR 1.3 [0.8–2.1]), nor when including survivors of refractory OHCA only (OR 0.5 [0.0–5.8]).

## Discussion

In the current study of consecutive OHCA patients with either refractory OHCA or prehospital ROSC, we found a lower crude survival rate in refractory OHCA patients but no difference in neurological outcome of survivors between the two groups of patients. Prehospital OHCA circumstances differed between the two groups, with prehospital ROSC patients more frequently having OHCA in a public setting, more often witnessed by the EMS, and with a shorter time to ROSC, whereas time to EMS arrival did not differ. Time from emergency call to the ED was longer in prehospital ROSC compared with refractory OHCA, indicating that a load-and-go approach may have been used for selected patients in the refractory OHCA group. The use of load-and-go is not documented on the Utstein-style registration sheet; however, an estimation of load-and-go can be made by looking at time from EMS arrival to arrival at the ED. Taking the short distances to hospital, characteristic of our region, into account, a load-and-go may be presumed when time from EMS arrival to arrival at the ED is less than 20 min. Using this approach, we found that a load-and-go approach may have been used in up to 33% of all refractory OHCA cases, though missing data for this parameter was high (60%). Lower age, public or witnessed OHCA, shockable rhythm, and later study year (calendar year) were independently associated with transportation to hospital instead of prehospital termination of the resuscitation attempt in refractory OHCA. This is in agreement with previously recommended advanced life support (ALS) termination of resuscitation rules, which encourage considering the possibility of terminating resuscitation when OHCA is not witnessed, no bystander CPR is provided, no ROSC despite ALS in the field, and no automated external defibrillator shocks are delivered [19]. We found no difference in etiology or postresuscitation care between the two groups, except in the number of patients admitted to ICU, with all refractory OHCA patients being admitted. Younger age, public or witnessed OHCA, bystander CPR, lower comorbidity burden, and shorter resuscitation length were all significantly associated with a higher 30-day survival rate.

Interestingly, despite lower survival rate in refractory OHCA patients than in prehospital ROSC patients, neurological outcome was similar, with more than 80% of the patients surviving to discharge with a favorable neurological outcome. This is in contrast to an earlier report where extensive cerebral damage was found to be frequent in survivors of refractory OHCA [23], suggesting that both pre- and in-hospital treatment of OHCA and postresuscitation care have improved during the last few decades. In continuation of this, it is worth noting that although later study year was associated with transportation to hospital with refractory OHCA, it was not associated with 30-day mortality. That is, though a greater proportion of refractory OHCA patients were transported to hospital with ongoing CPR, 30-day mortality was unaffected, indicating a relative increase in the number of patients surviving after refractory OHCA, which may be attributed to a combination of improved treatment possibilities and optimal selection of patients to transport with ongoing CPR. Also, Herlitz et al. [23] did not report time to ROSC, and differences in this factor between their study and ours might contribute to the differences in neurological outcome. Also of interest, reason for termination of active in-hospital treatment was more often anoxic brain damage in patients with prehospital ROSC than in refractory OHCA, which may indicate a reasonable selection bias in refractory OHCA candidates for transportation by the EMS system.

Favorable neurological outcome was equally high in the two groups despite the difference in time to ROSC (median 15 and 27 min, respectively). By extension, although resuscitation length > 30 min was associated with lower 30-day survival, it was associated with neither better nor worse neurological outcome. A prior study, however, found contrary results, with duration of CPR inversely associated with favorable neurological outcome 30 days post-OHCA [6]. This discrepancy in the association between resuscitation length and neurological outcome might be a result of differences in the study method; the current study included survivors only (CPC 1–4), whereas Goto et al. included nonsurvivors in their study (CPC 5). A low patient number in the current study or regional differences between Denmark and Japan [15] should also be considered as a possible cause of the discrepancy. Nevertheless, this study indicates that long resuscitation attempts despite refractory OHCA are not in vain, and those surviving in general achieve a favorable neurological outcome. This is partially supported by Nagao et al., who found that prehospital resuscitation efforts to achieve favorable neurological outcome should be continued for at least 40 min in patients with bystander-witnessed OHCA [24]. By extension, we found that 31% of patients with refractory OHCA and duration of resuscitation > 40 min achieved ROSC, and 14% of refractory OHCA patients achieved ROSC despite duration of resuscitation > 60 min (Fig. 2b). This, together with a good neurological outcome in 86% of all refractory OHCA survivors, indicates that the minimum duration of resuscitation in case of OHCA should be set even more conservatively than the one of 40 min suggested by Nagao et al. Of note, both a substantially greater study population and a lower percentage of good neurological outcome (70% in the bystander-witnessed OHCA group when calculated as in the current study) together with regional differences could account for the discrepancy between the results.

In patients achieving and/or having sustained ROSC in the ED only, 39% of patients with refractory OHCA and 46% of patients with prehospital ROSC were still alive after 30 days. The main reason for this difference is probably time to ROSC; the inverse association that we found between length of resuscitation and 30-day survival supports this. Of note however, the association we found might be overestimated when taking into consideration that more patients with refractory OHCA had treatment terminated because of time to ROSC, thereby possibly creating a bias. With regard to postresuscitation care, no difference other than number of patients admitted to the ICU was found, indicating that more patients in the refractory OHCA group required highly specialized treatment or were selected to have maximum postresuscitation care treatment. Looking into prehospital OHCA circumstances, however, we found that more patients with prehospital ROSC had favorable circumstances, with a higher degree of OHCA in public and more with EMS-witnessed OHCA. In line with this, both public and witnessed OHCA were associated with 30-day survival and might account for some of the difference in survival between the two groups.

A point of action to increase survival after OHCA is to decrease the period of low flow specifically. Efforts in this area are ongoing with the implementation of eCPR during either OHCA or in-hospital cardiac arrest on a trial basis worldwide [10–13, 25]. Retrospective and prospective studies indicate that eCPR increases survival in selected patients [14, 25]. Our result of NNT for refractory OHCA patients of 4.9 and 121 when also including OHCA patients with treatment terminated in the prehospital setting indicates that eCPR might be beneficial for the selected group of refractory OHCA patients (defined as patients transported to hospital with ongoing CPR), whereas the cost-to-benefit ratio of including all patients, unselected, might not be advantageous. One retrospective study indicates that door-to-implantation time of eCPR is critical and found that implantation in < 30 min improved 30-day survival in refractory OHCA [26]. It is interesting that more patients with refractory OHCA compared with patients with prehospital ROSC had treatment terminated because of circulatory failure. Could eCPR have made a difference for these patients? However, no randomized controlled studies on the use of eCPR in OHCA have yet been completed, and the potential of eCPR to increase survival in an OHCA population is still not certain. Likewise, it is uncertain how eCPR will affect neurological outcome in an OHCA population.

eCPR is currently being introduced in Denmark, and a national register has just recently been established to record mortality and morbidity after OHCA treated with eCPR. In this field, the definition of the denominator is very important when reporting outcomes representing either all OHCA patients with attempted resuscitation, patients brought to hospital with refractory OHCA, or patients with ROSC and cardiogenic shock. The current study of a cohort of OHCA patients without eCPR treatment attempts to establish a baseline for future studies, preferably randomized controlled trials, on the effect of eCPR on mortality and morbidity.

### Limitations

Study limitations include the retrospective, observational design with a fairly low number of patients with refractory OHCA. Unfortunately, when looking at resuscitation time, we had about 200 missing values (14%); this probably accounts for differences in all survival times when using this parameter as opposed to refractory OHCA vs. prehospital ROSC when analyzing survival. Use of automated external defibrillators would have been an interesting variable to look into; no data on this were available, however, but previous studies have found their use to be limited in the study years included in the current study [1]. A load-and-go approach when distance to nearest hospital, and thereby advanced treatment, is short might decrease the no- or low-flow period during refractory OHCA. Unfortunately, we did not have sufficient data to investigate whether distance to hospital was associated with chance of transport to hospital with ongoing CPR (load-and-go), nor did we have sufficient data on the association with survival or neurological outcome. Of note, the EMS system in the Capital Region of Denmark with a prehospital physician might not be fully generalizable to other regions/countries. Likewise, a relatively short distance to hospital throughout this region and a high percentage of bystander CPR in Denmark in general might explain the relatively short time to ROSC reported in this study, even for patients with refractory OHCA. A certain degree of selection bias might have occurred in the prehospital setting, resulting in more patients with favorable circumstances, such as short distance to hospital, being included in the refractory OHCA group because of a load-and-go approach. This is supported by Fig. 2, where a resuscitation time less than 20–30 min could indicate load-and-go.

## Conclusions

Thirty-day survival after refractory OHCA with ongoing CPR at hospital arrival was 20% compared with 42% in patients with prehospital ROSC. Though prognosis was less favorable in patients with refractory OHCA, the vast majority of survivors were discharged with a favorable neurological outcome. Also, we found an increase in the number of patients surviving refractory OHCA through the study period, this despite a conservative postresuscitation care approach without the use of eCPR. The present study indicates that prolonged resuscitation of refractory OHCA is not in vain and that patients surviving the first month post-OHCA achieve a good neurological outcome similar to patients with prehospital ROSC, making implementation of eCPR to increase survival of refractory OHCA even more interesting. Randomized studies on survival and neurological outcome in connection to refractory OHCA managed by eCPR are necessary.

## Abbreviations

ALS: Advanced life support
CABG: Coronary artery bypass grafting
CAG: Coronary angiography
CCI: Charlson comorbidity index
CPC: Cerebral Performance Categories scale
CPR: Cardiopulmonary resuscitation
CT: Computed tomography
eCPR: Extracorporeal cardiopulmonary resuscitation
ED: Emergency department
EEG: Electroencephalography
EMS: Emergency medical services
IABP: Intra-aortic balloon pump
ICD: Implantable cardioverter defibrillator
ICU: Intensive care unit
LVEF: Left ventricular ejection fraction
NNT: Number needed to treat
OHCA: Out-of-hospital cardiac arrest
PCI: Percutaneous coronary intervention
ROSC: Return of spontaneous circulation
STEMI: ST segment elevation myocardial infarction
TTM: Targeted temperature management
